# Decontamination of *Escherichia coli* on dried onion flakes and black pepper using Infra-red, ultraviolet and ozone hurdle technologies

**DOI:** 10.1016/j.heliyon.2021.e07259

**Published:** 2021-06-07

**Authors:** Nada El Darra, Fei Xie, Prashant Kamble, Zakir Khan, Ian Watson

**Affiliations:** a Beirut Arab University, Faculty of Health Sciences, Tarik El Jedidah – Beirut, P.O.Box: 115020 Riad El Solh 1107 2809, Lebanon; b Systems, Power and Energy Research Division, School of Engineering, University of Glasgow, James Watt (South) Building University Avenue, Glasgow, UK; c Department of Chemical Engineering, COMSATS University Islamabad, Lahore campus, Lahore, 54000, Pakistan

**Keywords:** Infra-red, IR, Ultraviolet, UV, Ozone, Combination, Treatment, Onion flakes, Black pepper, Decontamination, Inactivation, *E. coli*, Synergistic, Spices

## Abstract

Among spices, onion flakes (OF) and black pepper (BP) are commonly used ingredients in domestic cooking; however, spices have been shown to be highly contaminated with pathogenic bacteria and bacterial spores. A novel method applying a treatments of Ozone Ultraviolet (UV, 255nm) and Infra-red (IR) light in different combinations was assessed for its efficiency in decontaminating OF and BP. In this study, untreated samples, as purchased, were inoculated with 9.5 × 10^5^ cfu/mL *Escherichia coli* (MG1655) and exposed to each treatment alone and in combination of ozone sequentially followed by UV/IR, and UV/IR combined followed sequentially by ozone. A difference in response towards the treatment was shown among the types of spices, with a high efficacy for BP. Typically 3 log reductions were observed for ozone, UV and IR. The sequential treatments of ozone with UV and IR combined gave improved results than individual ones, with 99.99% of *E. coli* inactivation, and a shorter exposure duration with ozone (2.5 and 5 min) and UV and IR (2.5 and 5 min). The combined effect (ozone 2.5 min, UV and IR 10 min) yielded a log reduction of 2.69 and 4.20 for OF and BP respectively, greater than the additive effect of the individual treatments alone. The IR lamp was modulated to reduce excessive temperature rise. This novel prototype was shown to be very effective in decontaminating spices. Further studies should be conducted to validate the effectiveness of this method on decontamination of various bacterial strains.

## 1 Introduction

Although spices generally have a low moisture content, which reduces microbial growth, their contamination has contributed significantly to food-borne infections and outbreaks (Al Bayssari et al., 2014; Banach et al., 2016). Spices have been shown to be a vehicle of chemical (mycotoxins) and microbial contamination (pathogenic bacteria like salmonella), while the actual cause of contamination is often difficult to determine (Federal Institute for Risk Assessment, 2016). Among spices, dried onions are widely used in the food industry, especially in manufacturing sauces, soups and salad dressings (Brown and Jiang, 2008; Ye et al., 2013). They are present in retail markets and they are available under different forms such as powder or flakes, and packed or unpacked. As they are widely available, their use as an ingredient in domestic cooking has increased. Onion powder is also considered to be a nutraceutical because of its antimicrobial, anticancer, and other health-promoting activities, owing to their sulfur, phenols, flavonoids, and selenium content (Corzo-Martínez et al., 2007; Pandurangan et al., 2016).

As with any other agricultural products, onions can be exposed to contamination at any stage of the food chain, starting from harvesting till the product is dispatched. Research studies have found that onion powder is susceptible to contamination with many potential food borne pathogens, such as *Bacillus cereus*, *Salmonella*, *Escherichia coli*, *Clostridium perfringens* and toxigenic moulds (McKee, 1995; CDC (Centers for Disease Control and Prevention, 2017)). Brown and Jiang (2008) found that onion powder samples were contaminated with tetracycline and ceftriaxone-resistant bacteria, this has important implications since these antibiotics have been used extensively in human, animal, and agricultural applications.

Several techniques have been tested for their efficiency in decontaminating powdered or flaked food including ultraviolet (UV–C) light treatment, chemical sanitizers, γ-irradiation and superheated steam treatment (FARKAS, 2001; Alfy et al., 2016). However, some techniques were shown to affect the quality and acceptance of spices due to their drying effect on aromatic compounds, deterioration of color and loss of nutrients or due to consumer unacceptability in the case of irradiation (Fine and Gervais, 2004; Rico et al., 2010). Pezzutti et al. (2005) tested the efficacy of gamma ray doses from 5 to 25 kGy on decontamination of onion powder (Pezzutti et al., 2005). They found that an application of 5 kGy was sufficient to decontaminate the powder from sulfite-reducing *Clostridia* and their spores, *B. cereus* and their spores, moulds, yeast and total coliforms. Brodowska and Śmigielski (2013) tested the efficacy of ozonation in decontaminating onion flakes. The results revealed that ozonation reduced *Enterobacteriaceae* counts, but there was no noted effect on total mesophilic bacteria or total fungal count (Brodowska and Śmigielski, 2013). Kim and Min (2018) tested the effect of cold plasma techniques on decontaminating onion flakes. A plasma frequency of 15 kHz for 20 min, combined with moisture vaporization, achieved the greatest pathogen reduction (reduction of 1.4 log CFU/cm^2^), compared to the other CP treatment using helium for 2–20 min at 15–35 kHz, without altering their physicochemical properties (including surface morphology, color, moisture content, and ascorbic acid and quercetin concentrations), although this reduction was not able to statisfy the food safety standards.

Black pepper (*Piper nigrum* L., BP) is also a widely used, valuable ingredient, known for its distinctive odor and flavour (Nisha et al., 2009). De Boer et al. (1985)^2020^, assessed the microflora of 150 samples of 54 different spices, spices mixtures and herbs from retail suppliers and found black pepper to be highly contaminated with *salmonella, Aspergillus* and *Penicillium* species, as well as spore forming bacteria such as *Bacillus cereus* and *Clostridium pefringens*. Clostridia and Bacillus are soil borne so form part of the natural microflora of black pepper. The contamination could be due to violation of hygienic handling practices during the drying process, consequently causing foodborne diseases. This is particularly a problem with black pepper (BP) as it is often applied directly on to the food in the raw state, without any heat treatment or cooking (Little et al., 2003). Based on the RASFF (Rapid Alert System for Food and Feed) summary reports, *Salmonella spp* was shown to be the most reported pathogenic microorganism in spices, with BP presenting 8% of the contamination (Banach et al., 2016). The high prevalence of *salmonella* in BP, added to salami, caused an outbreak in 44 states of the United States of America in 2010 (Gieraltowski, 2013).

Several decontamination techniques have been assessed for their efficacy to treat BP. Gamma irradiation treatment and ethylene oxide fumigation were shown to be quite efficient. However, ethylene oxide was banned by the European Union due to its carcinogenic effects (Tateo and Bononi, 2006). Gamma irradiation can be applied in controlled doses but presents questionable consumer acceptability (Trindade et al., 2009). Sádecká et al. (2004) have proved that a dose of 5 kGy of ionizing radiation was sufficient to decontaminate BP from microbial contamination, with no significant change in volatile oil compounds (Sádecká et al., 2004). Steam treatment was also applied to BP but it was shown to be effective only with low microbial loads; for higher loads, only a low reduction in contamination was achieved and the aroma and odors were possibly alterated (Schweiggert et al., 2007). Pulsed UV light is another technique that has been tested for decontamination of BP. This method was shown to achieve less than 1 log reduction against *Saccharomyces cerevisiae* with rapid modification of color (Fine and Gervais, 2004). Hertwig et al. (2015) evaluated the performance of cold atmospheric pressure plasma and a reduction of 2.8–4.1 log was noted for pathogenic bacterial (*S. enterica*, *B. subtilis, B. atrophaeus*) spores tested. After 30 min of remote plasma treatment, no considerable effect on the quality parameters was observed (Hertwig et al., 2015). Erdoğdu & Ekiz (2013) tested the combined effect of far infrared (FIR (wavelength not specified), 650 W) and ultraviolet (UVC) radiation on surface pasteurization of BP seeds. A significant reduction of total mesophilic aerobic bacteria was noted for FIR after 4.7 and 3.5 min at 300 and 350 °C, respectively. However, UVC alone or combined with FIR, did not show a significant bacterial reduction.

Currently, there are only relatively ineffective methods for decontamination of OF and BP available and alternative strategies are needed that are consumer friendly. Few studies have assessed the efficacy of combined systems to decontaminate BP and OF. Therefore, the aim of the present study was to assess the performance of a system combining UV-C radiation and modulated IR light sequentially with ozone, developed in our previous work (Watson et al., 2019), on decontamination of OF and BP, artificially inoculated with *Escherichia coli* as a test organism. The treatments are performed in a fludised bed, and include the treatments alone (Ozone, UV, and IR) and in combination (ozone then UV combined with IR; or UV combined with IR followed by ozone treatment).

## 2 Materials and methods

### 2.1 System description

**Figure 1:**
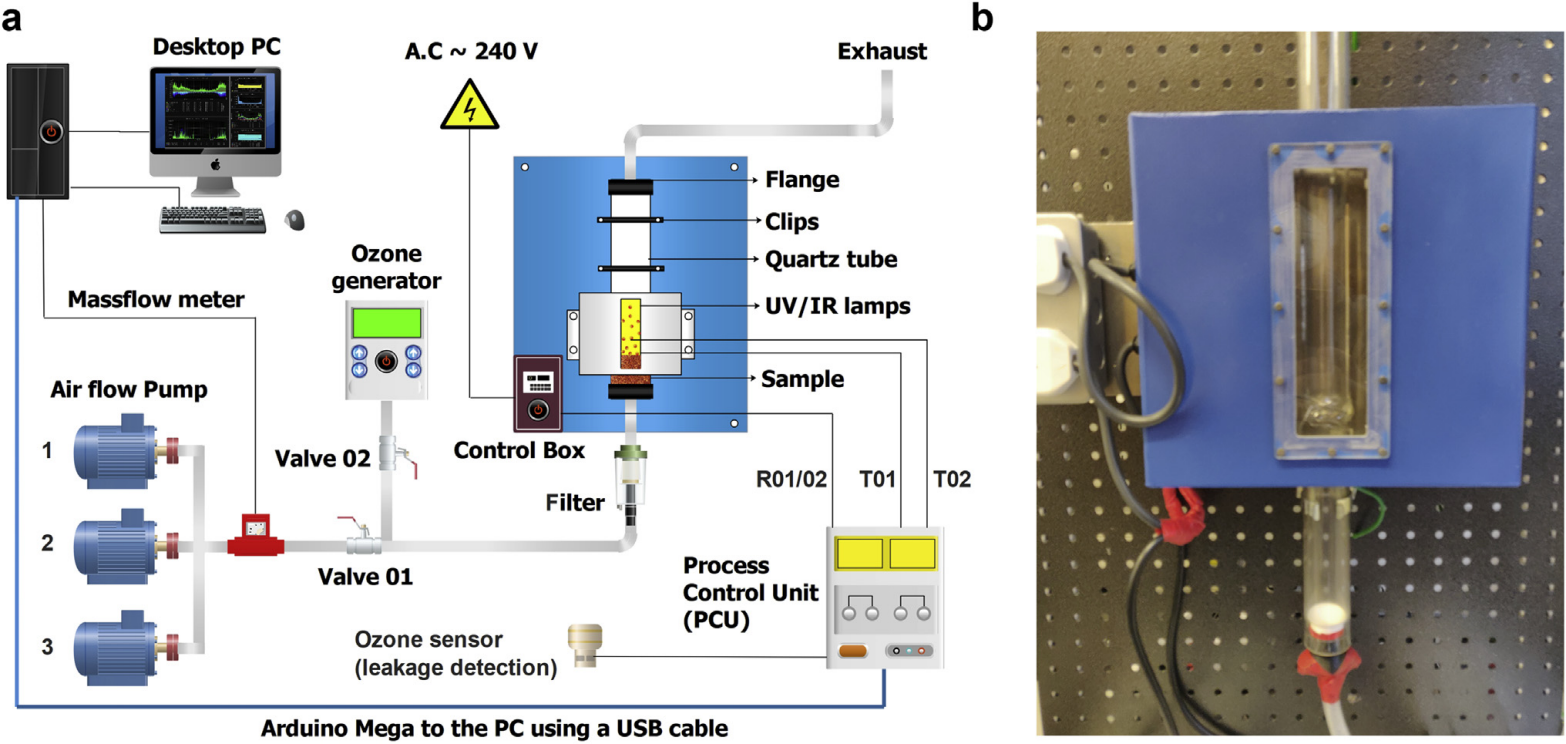
(a) Schematic of the experimental set up of the combined ozone, UV, IR and decontamination system (b) Photograph of the experimental set up showing the decontamination chamber (blue box 49 cm wide by 82 cm high).

**Figure 2:**
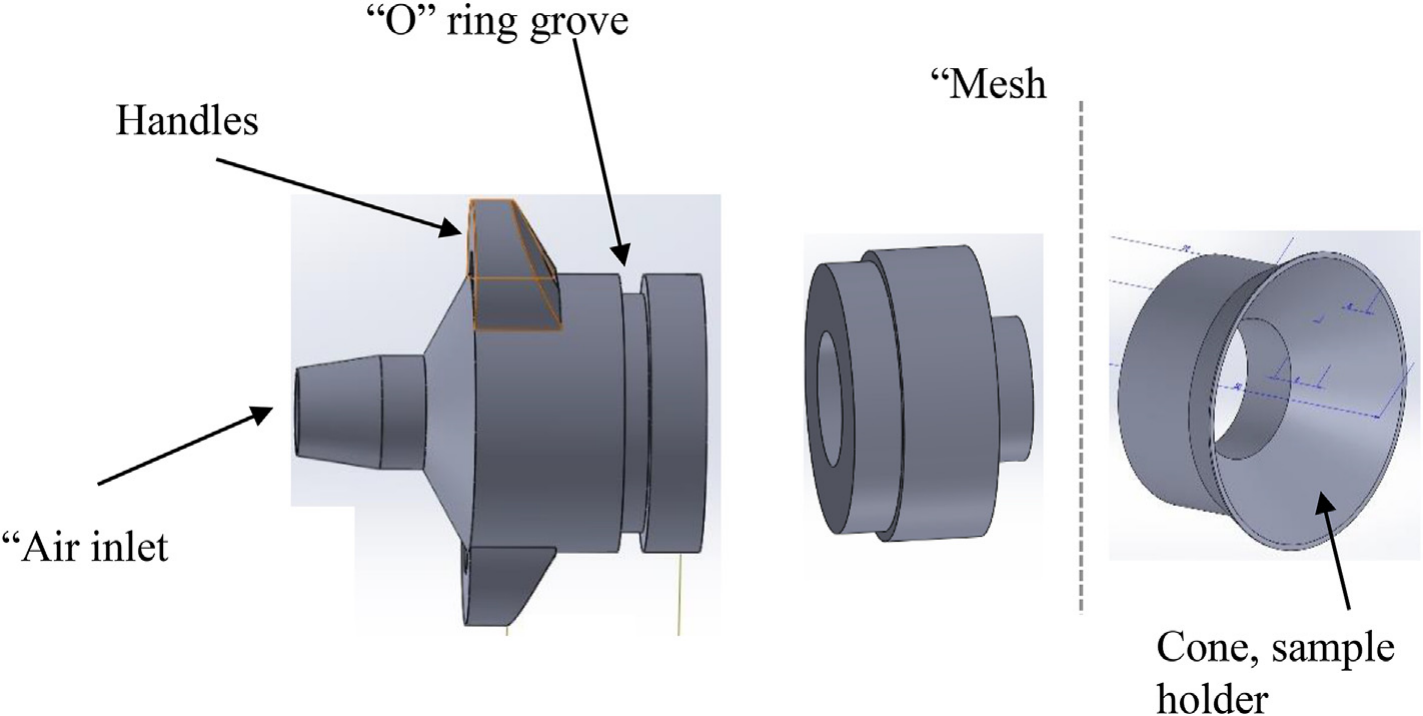
Rapid prototyped quartz tube insert; left image, bottom component, showing air inlet, handles and “O” ring groove; middle, clips into the bottom and top component with the mesh between the middle and right component, which shows the sample holder and cone to collect samples as they fall down the quartz tube.

The system (schematic shown in Figure 1a and photo in 1b) was slightly modified from Watson et al. (2019); the fused quartz tube dimensions remained the same (900-mm long, mounted vertically, with an inside diameter (ID of 33 mm and wall thickness of 3 mm)). Three air pumps (MA100-120, Jecod, UK) provided an air flow rate of up to 120 L/min which reduced to 92.25 ± 2.26 L/min (n = 19 readings) in the tube, the flow rate was controlled with a mass flow meter (Red-Y, Vogtlin, Germany). Attached at the lower end of the quartz tube was a plastic, rapid prototyped component, comprising of three sections that could be pushed together; this component, or tube insert, sealed the tube, conditioned the air flow and was the sample container. Figure 2 shows the 3 components that make up the tube insert, these are: left) the bottom part with an air inlet, small side handles for easy insertion and removal and an “O” ring groove that allowed the tube to be sealed; middle) which clips into the bottom and the top part, as seen in the right image) which shows where the samples were aseptically placed. The conical shape was designed to allow the BP and OF to conveniently fall back into the air flow. The distributer plate and assembly were designed to suspend the OF or BP in the even air flow over the cross-sectional area of the tube. Calculations were done previously (Watson et al., 2019) to determine the distributor plate hole size using fluidized bed theory and in this case a mesh was used which prevented the OF or BP falling down the air tube and allowed a uniform velocity such that the BP or OF would rise and fall in the tube when the velocity was above a minimum threshold of ~92 ms^−1^. The tube insert allowed the OF/BP samples to be placed inside the tube aseptically, and the insert was simply cleaned each time.

The lighting, a standard germicidal (UV, 4 W, effective length 80 mm, 254 nm) and IR lamp (100 W, SK15 Jacketed, Victory Lighting, UK, colour temperature 2550K)) remained the same as in the first experiments on chilli flakes (Watson et al., 2019); the lamps were placed symmetrically opposite each other, approximately 25 cm from the base of the tube. The outside of the UV and IR tubes were placed 10 mm from the external part of the fused quartz tube, the effective length of the UV tube was 80 mm and for the IR lamp it was 180 mm. They were safely enclosed by a metal cover with a polycarbonate window (26.5 × 25 cm, see Figure 1b). The UV and IR lamps were controlled separately (on/off).

The outside surface temperature of the tube was measured with a thermocouple (TEMPer1F, PC Sensor, China); this temperature measurement was used to automatically modulate the IR lamp between about 58-62 °C; the sensor was placed away from direct IR exposure. As with the previous work on chilli flakes (Watson et al., 2019), the ozone treatment (GMB-DOM-024, GMB Ozone purifier, Italy; 300 mgO_3_hr^−1^, 25 W power consumption), was applied separately followed by the UV and IR treatment and on a loosely packed, stationary bed (i.e. without any air flow); this was because the ozone flow rate (2–3L/min) is much less than the air flow rate (120 L/min) and the ozone would be diluted to low levels in an air flow. The ozone flow into the system was controlled via an isolating valve.

The UV and IR lamps position relative to the OF/BP was easily modified by adjusting the tube up or down or by varying the air velocity. For a distance of about 25 cm from the bottom of the tube, the required air flow was about 92 L/min for the flakes to spend most of their time within the irradiated region.

The temperatures inside (middle) and outside of the tube were measured, in a fixed place, whilst automatically modulating the IR lamp.

### 2.2 Raw materials

OF and whole BP were purchased from a Lebanese supermarket (packaged in Avignon, France) and were evaluated for their microbiological loads,. The OF and BP were kept in a dry place at room temperature. Sterile water (9 mL) was added to 1 g samples of OF and BP, each were shaken for 20 s then followed by a serial dilutions (down to 1 in 10^6^). Afterwards, 20 μL samples were plated on Luria Bertani (LB) and incubated at 37 °C overnight (Sezonov et al., 2007). Consequently, the Colony forming Units were counted and the bacterial concentration calculated per gram, using Miles and Misra Method (or surface viable count) (Hedges et al., 1978). This analysis was done twice.

### 2.3 Microbiological investigation of spices samples

OF and BP microbiological parameters were analyzed according to international standard methods as shown in the Table 1, for the occurrence of: total mesophilic bacteria *Enterobacteriaceae, Total coliforms*, *Escherichia coli*, *Coagulase-positive staphylococci*, *Sulfite-reducing bacteria*, *Clostridium perfringens*, *Listeria monocytogenes*, *Salmonella* and *Yeasts and molds.* Microbial enumerations were expressed as log of colony forming unit per gram sample (CFU/g). The samples were compared with the Lebanese Standards Institution (LIBNOR) standards of OF and BP (http://www.libnor.gov.lb, 2002, 1999). LIBNOR is a public institution attached to the Ministry of Industry, Lebanon; it has the sole right to prepare, publish and amend national standards.

**Table 1:** Microbiological parameters investigated and relative identification techniques.

Microbiological parameters	ISO Reference Analytical method	Incubation conditions
*Total mesophilic bacteria*	ISO 4833:2013	30 °C
*Enterobacteria*	ISO 21528:2004	37 °C
*Total coliforms*	ISO 4832:2006	37 °C
*Escherichia coli*	ISO 16649-2:2001	44 °C
*Coagulase-positive staphylococci*	ISO 6888-1:1999	37 °C
*Sulfite-reducing bacteria*	ISO 15213:2003	37 °C
*Clostridium perfringens*	ISO 7937:2004	37 °C
*Listeria monocytogenes*	ISO 11290-1:1996	37 °C
*Salmonella*	ISO 6579:2002	37 °C
*Yeasts and molds*	ISO 21527-2008	25 °C

### 2.4 Sample inoculation

An overnight culture of of *Escherichia coli* (MG1655), a non-pathogenic strain (Stromberg et al., 2018), was supplied by the Environmental Engineering Laboratory, James Watt School of Engineering, at the University of Glasgow for inoculation of the OF and BP samples.

**Figure 3:**
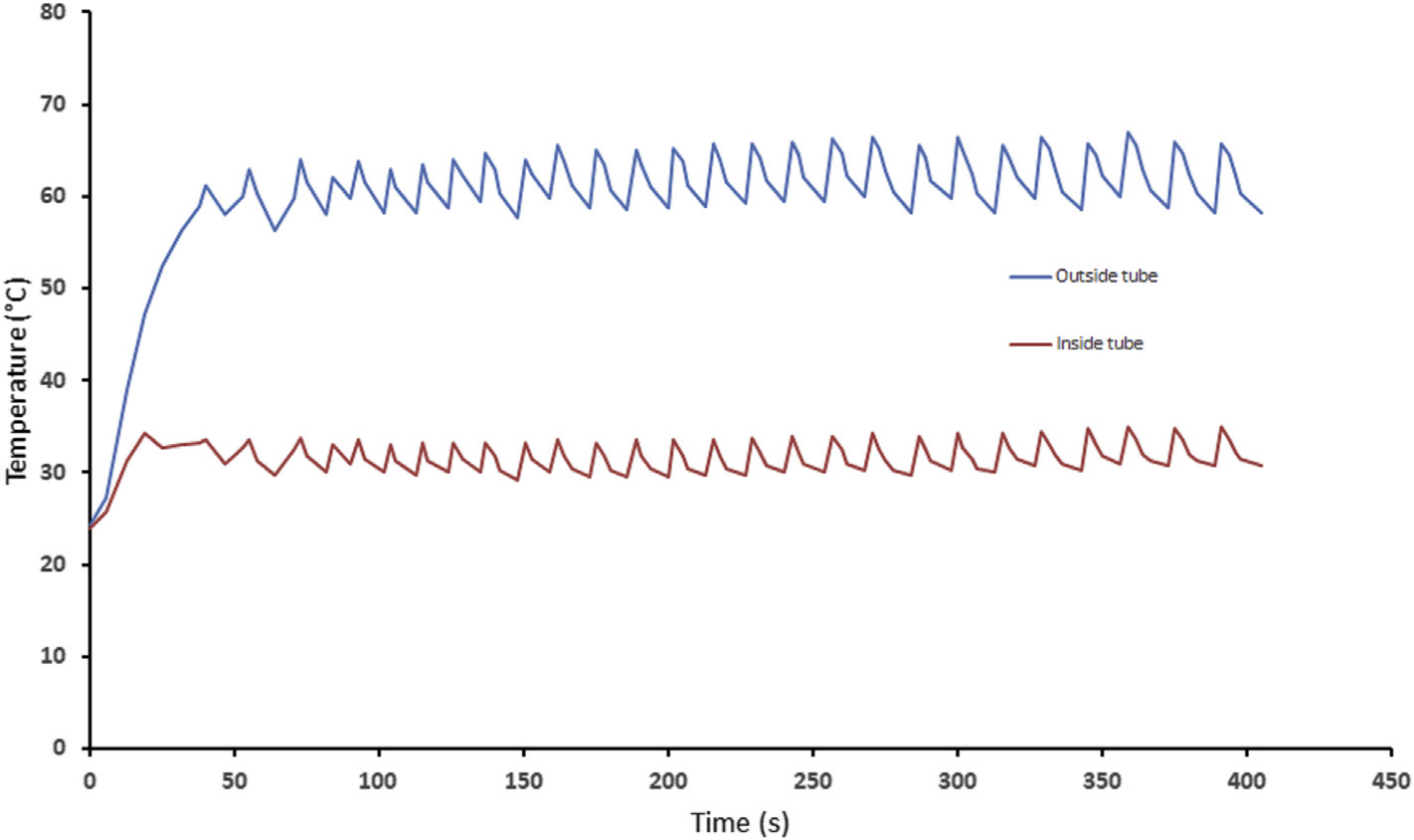
Centre temperature inside the tube (bottom) and external temperature (top) as a function of time, with IR modulation to maintain 58-62ºC temperature outside the tube, air flow rate of 120 L/min (temperature variation, SD ≤± 4ºC, n = 3).

Fifteen-mL of the *E. coli* overnight culture was pipetted onto 60 g of the “as purchased” OF and BP and mixed thoroughly; samples (3 × 1g) were analysed for inoculation efficiency immediately. The samples were then dried with the same conditions as Watson et al. (2019). Samples were taken every 30 min over the 2 h drying time to evaluate the reduction of the bacterial load. The initial microbial loads at time zero of the inoculated *E. coli* on OF and BP samples were 1.1 × 10^4^ and 1.35 × 10^4^ cfu/mL respectively. One gram of the inoculated OF or BP samples were placed into the sample holder (Figure 3) and a treatment was applied. After treatment, the sample was removed, added to a 9 mL sample of of sterile water and appropriate serial dilutions were made. This step was done in duplicate. The sample treatments are detailed below.

### 2.5 Sample treatments

The dried samples of OF and BP were exposed to different individual treatments: ozone, UV, IR and combinations of the 3 treatments. The individual treatment times for ozone, UV and IR alone were 0, 5, 10, 12.5, 15, 17.5, 20 and 30 min. Different combinations were tested; this included 1) Ozone for 2.5 and 5 min, each followed by a combined treatment of UV and IR for 2.5, 5, 7.5 and 10 min for the inoculated and raw OF and BP; and 2) A combined treatment of UV and IR for 2.5 and 5 min, each followed by ozone for 2.5, 5, 7.5 and 10 min.

It should be reiterated that with the ozone treatments the main flow line for air was not activated because of the flow differentials and in this system design it was not possible to combine ozone with the UV or IR simultaneously. The ozone treatment was then on a loosely packed, fixed bed of the OF and BP.

### 2.6 Statistical analysis

This work was designed to compare the relative efficiency of different treatment durations from Ozone, UV and IR and large datasets were not generated. As the work was done in duplicate, to improve the analysis the averages from each data point were compared statistically. Furthermore, it should be noted that the treatment levels are somewhat arbitrary and were chosen so that the death kinetics could be investigated for single and sequential/combined experiments. In this context, similar levels of decontamination are sought for each treatment, reducing their statistical differences. The F-test was used to compare the differences between the log reductions (CFU/g) for OF and BP for each treatment alone (Ozone, UV and IR) across all values. The t-test was used to compare Ozone with UV, Ozone with IR and UV with IR for OF and BP.

For treatments with i) ozone then UV and IR or ii) UV and IR first then ozone, the t-test was used for analysis between the OF and BP for the same treatment combination, and between the same sample (OF or BP) for UV/IR first with 2.5 and 5 min and for Ozone at 2.5 and 5 min followed by UV and IR.

## 3 Results and discussion

### 3.1 Microbial loads of the onion flakes and black pepper samples

The microbial load of untreated OF and BP samples were examined considering the LIBNOR norms 2002:614 and 1999:70, for OF and BP, respectively (see Table 1). From Table 2, the results indicated that the total mesophilic aerobic bacteria for OF and BP are 8.8 × 10^4^ and ~10^2^ CFU/g respectively, which is within LIBNOR maximum permitted limits. Tables 1 and 2 shows that *E. coli* was within the microbiological specifications of LIBNOR norms for OF and BP. No *Clostridium pefringens* and *Salmonella* were detected. Yeast and moulds were within the LIBNOR norms for OF and BP, which are 10^2^ and 5 × 10^2^ CFU/g, respectively. Overall, the OF and BP were within Lebanese norms. Consequently, the samples were inoculated with *E. coli* to identify the effect of the combined system ozone, UV, IR and combinations without any pre-treatment. This differed from the groups previous work (Watson et al., 2019), where the samples were inactivated before inoculation using microwave, autoclaving and ethanol treatment because of the high levels of contamination.

**Table 2:** Microbiological load of Onion flakes and Black pepper samples in (CFU/g), compared to the Lebanese standard LIBNOR norms for OF and BP.

Microbiological parameters	Onion flakes (OF)	LIBNOR NORM 2002:614 for OF	Black Pepper (BP)	LIBNOR NORM 1999:70 for BP
*Total mesophilic bacteria* (CFU/g)	8.8 × 10^4^	10^5^–10^6^	10^2^	10^5^–10^6^
*Enterobacteria* (CFU/g)	0	Not regulated	0	Not regulated
*Total coliforms* (CFU/g)	<10	Not regulated	<10	Not regulated
*Escherichia coli* (CFU/g)	<10	10–10^2^	<10	10–10^2^
*Coagulase-positive* *staphylococci* (CFU/g)	0	Not regulated	0	Not regulated
*Sulfite-reducing bacteria* (CFU/g)	<10	Not regulated	<10	Not regulated
*Clostridium perfringens* (CFU/g)	0	10–10^2^	0	10–10^2^
*Listeria monocytogenes* (CFU/g)	0	Not regulated	0	Not regulated
*Salmonella* (CFU/25 g)	0	Absent	0	Absent
*Yeasts and molds* (CFU/g)	10^2^	10^3^–10^4^	5 × 10^2^	10^3^–10^4^

### 3.2 System overview

The variation of temperature between the inside centre of the tube and outside of the tube over 400 s with the flow on (~92 L/min) is shown in Figure 3. It is to be noted that the lamp was modulated on/off to maintain an external temperature between 58 - 65 °C. The temperature inside the tube was relatively low, being maintained between 29 and 34 °C. The temperature rises over the first 19 s period followed by a temperature stabilization at an average of 60 and 32 °C, for outside and inside the tube, respectively. These conditions are shown to preserve the organoleptic properties of OF and BP, since a fairly low temperature of 40–60 °C do not affect flavours (Haley and McDonald, 2016).

### 3.3 Drying kinetics of CF

**Figure 4:**
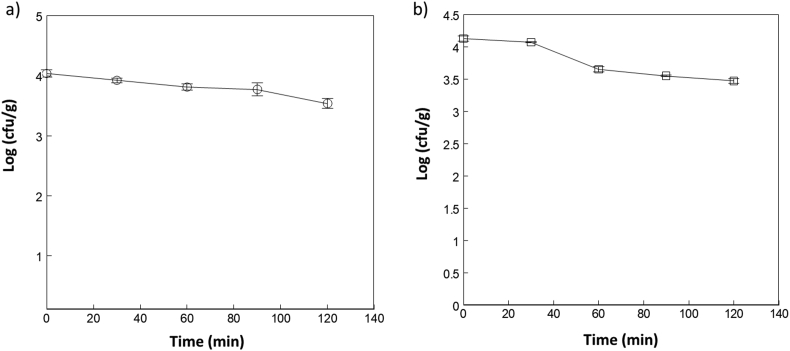
*E.coli* population kinetics, recovered log (cfu/g) from the inoculated Onion Flakes (OF, a) and Black pepper (BP, b) over the drying time (t=0, 30, 60, 90 & 120 min). Each point is the average of 3 readings.

After inoculation, the samples were dried in the oven until they were visibly dry. Figure 4a and b show the *E. coli* reduction curves during the drying processes at different times (t = 0, 30, 60, 90, 120 min) in duplicate, for OF and BP. The initial microbial loads at time zero of the inoculated *E. coli* on OF and BP samples were 1.1 × 10^4^ and 1.35 × 10^4^ cfu/mL, equivalent to log 4.04 cfu/g and log 4.13 cfu/g, respectively; representing a reduction in culturability of 68% and 78% over 2 h for OF and BP, respectively. This reduction is higher than those observed with Chilli Flake (CF) drying reported by Watson et al. (2019) which was 55%. The bacterial reduction on drying is likely due to the decrease in water activity, leading to sublethally injured cells which are difficult to recover as viable colony forming units (Bourdoux et al., 2016; Gurtler et al., 2019).

### 3.3 Individual treatment for inoculated OF and BP

**Figure 5:**
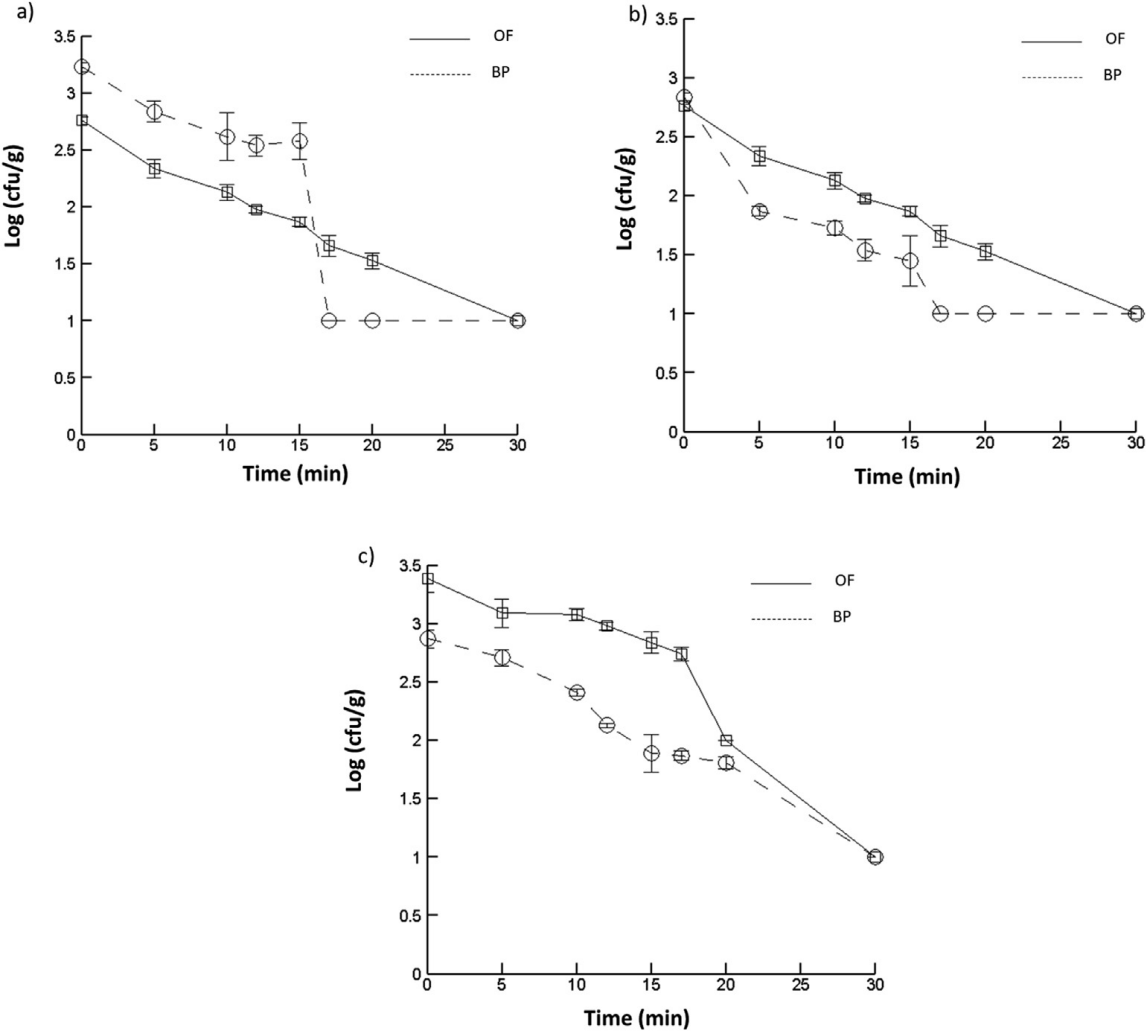
*E.coli* population kinetics, log (cfu/g) recovered from inoculated OF and BP after individual treatments by (5a) ozone for different durations (t=0, 5, 10, 12, 15, 17.5, 20 min) and (5b) UV and (5c) IR for different durations (t=0, 2, 5, 10, 20 min). Error bars, SD, n =2.

Figure 5 shows the log reduction in the bacterial counts of OF and BP for the individual treatments over 30 min, with (a) ozone treatment for different durations (t = 0, 5, 10, 12, 15, 17.5, 20 min) and (5b) UV and (5c) modulated IR for different durations (t = 0, 2, 5, 10, 20 min) controlled to maintain an external temperature of approximately 60 °C. As observed in the figures, the survival of microorganisms decreased with increasing treatment time for each process, as similarly found by Erdoğdu and Ekiz (2013) investigating IR and UV treatment of BP seeds. IR heating has produced promising results for the surface pasteurization of black pepper seeds (Erdoǧdu and Ekiz, 2013), as well as for cumin seeds if combined with ultraviolet treatment (Erdoǧdu and Ekiz, 2013). All the treatments produced complete reduction to the limit of enumeration of the *E. coli* inoculated on the OF ≤30 min and after 20 min of ozone, UV or IR treatment, a bacterial culturability reduction of 67%, 94% and 95% was observed, respectively. For BP (Figure 5), 20 min of either ozone or UV achieved complete inactivation of *E. coli*, and 30 min of IR were needed for complete inactivation. Ozone and UV treatments produced complete inactivation of the *E. coli* inoculated on the BP after 17 min. However, 20 min of IR was insufficient to achieve a complete inactivation, which given the internal temperature of the tube was only 32 °C is understandable. After 15 min of ozone, UV and IR a bacterial culturability reduction of 78%, 95% and 90%, respectively, was observed on black pepper. Given that for OF the reductions were 67%, 94% and 95% for the same treatments after 20 min, the efficacy of the ozone and UV were slightly higher with BP than OF. However, for OF, a treatment >20 and ≤30 min was required to achieve complete inactivation for all three treatments, from the data points taken.

A comparison of ozone application on OF and BP showed greater reduction on BP compared to OF. This is shown by 1 log reduction achieved for 17 min ozone treatment for BP and 30 min for OF, since 17 min of ozone for OF has led to 1.66 log reduction. This could be due to the fact that surface area is a critical factor for the efficacy of ozonation treatment, as mentioned in the study of Akbas and Ozdemir (2008) and the shape of the flakes verses the spherical nature of the black pepper. The inoculated bacteria could be more protected in flaked onions than BP, since the ozone power is affected by the penetration depth and the interation of ozone with organic materials (Akbas and Ozdemir, 2008). Similar results were obtained while comparing the efficacy of UV on OF and BP, with a better result on whole BP. This could be due to the fact that UV provides better results when used on geometrically round shapes (Lagunas-Solar et al., 2006).

It is worth noting, then, that the random flow of OF and BP allowed a larger UV dose to reach the different surfaces because of the turbulent mixing and a high decontamination effect of inoculated *E. coli* was observed. UV-C disinfection is influenced by various factors such as distance from the lamp, UV intensity, and exposure time (Yaun et al., 2004; Allende et al., 2006; Cha et al., 2010). The lethal effect of UV is due to an alteration of the cell replication, leading to cell death (Allende et al., 2006).

IR was less effective than the other treatments on both OF and BP, but still showed some inactivation, even given the relatively low temperature rise inside the flow tube. This may be due to rapid heat transfer mechanisms that reduce the bacterial load during a short process time (Erdoğdu & Ekiz, 2011, 2013), it should be noted that the samples are under forced convection conditions which may provide cooling.

### 3.4 Sequential treatments

**Figure 6:**
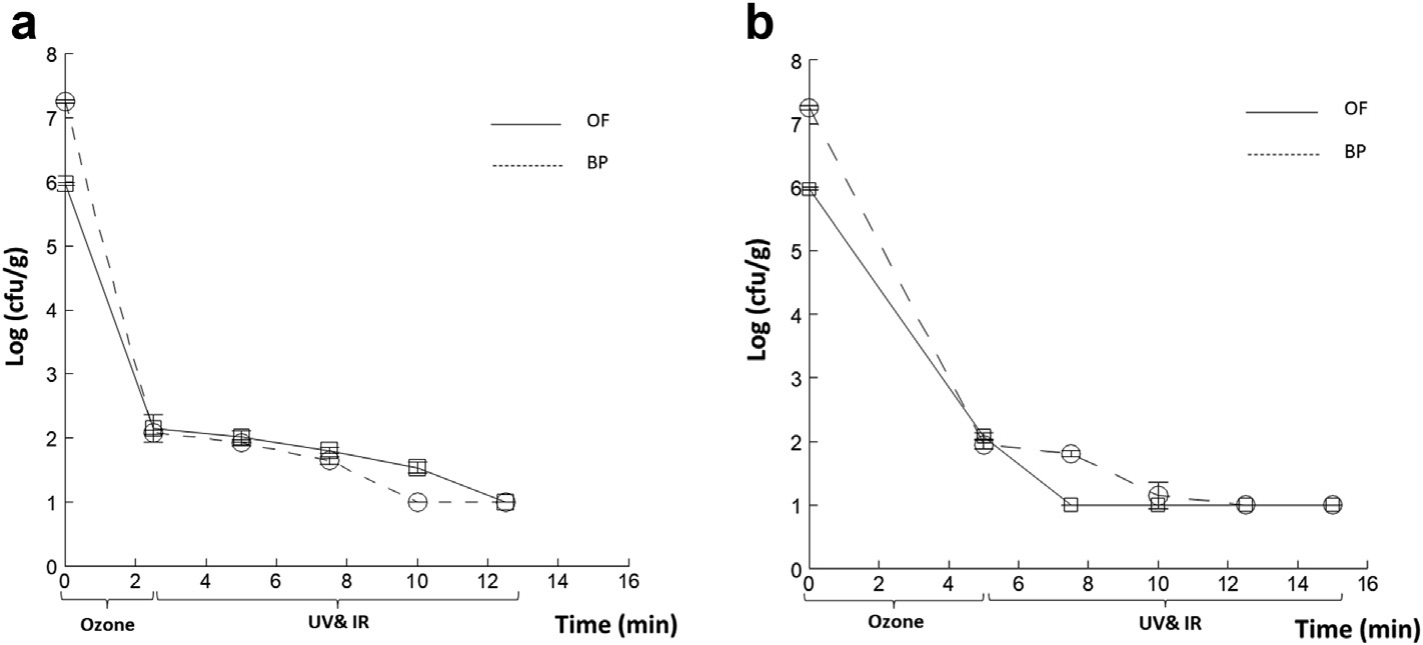
*E.coli* population kinetics of inoculated Onion Flakes (OF) and Black Pepper (BP), showing log (cfu/g), treated by ozone OF for 2.5 min (a), and 5 min (b) followed respectively by a combined treatment (UV & IR) for 2.5, 5, 7.5, 10 minutes. Error bars, SD, n =2.

Watson et al. (2019) previously studied the effects of combining ozone (20 min) followed by UV and IR (60 min) on the decontamination of CF inoculated with *E. coli*, here the ozone had a dominant effect, and the duration of the treatment was too long, i.e. the treatment time consistently exceeded the point at which no CFU were detected. Consequently, in this paper the exposure time was shortened for the ozone pretreatment by applying it for only 2.5 min (Figure 6a) and 5 min (6b) followed by UV & IR for 10 min for OF and BP. In this way it was anticipated that greater information would be obtained on the combined treatment effects post-ozone treatment. It is seen from these graphs that a reduction of 99.98 and 99.99 % for an ozone treatment of 2.5 min was observed for OF and BP, with a similar reduction of 99.98 and 99.99% for 5 min treatment time with OF and BP. Consequently, since the same response was obtained for these different periods of time, it makes sense to use the shorter treatment (2.5 min) as no benefit was observed with increased exposure. The single dose treatment curves, in Figure 5, show fairly low inactivation during the early stages of the ozone treatment, which is why there was not a substantial difference for 2.5 or 5 min. It is not clear why this is the case but it is likely that there is a threshold exposure needed to inactivate most of the microorganisms, with less robust ones inactivated with a lower dose or perhaps such microorganisms are in more accessible places for the treatment.

For OF inoculated with *E. coli*, a combined treatment of UV and IR for 2.5 min following the ozone 5 min exposure was sufficient to acheive complete inactivation (Figure 6). It is seen from these graphs that a reduction of 99.99 % for UV and IR treatment of 2.5 min was observed for both OF and BP, with a similar reduction of 99.90 and 99.99% for 5 min of UV and IR with OF and BP.

However, with 2.5 min ozone, 10 min of combined UV and IR were needed to reach the same result, noting that 30 min of ozone alone was needed to decontaminate OF. Combining ozone with UV and IR reduced the treatment time and increased its efficacy. For BP, 20 min of ozone alone was needed for decontamination. Combining treatment therefore has reduced the exposure duration. This result is in agreement with the previous work done on CF, which showed that combining treatments increases the decontamination efficacy through stimulating more damage to the cell envelope related to the synergistic lethal effect of the treatments (Watson et al., 2019). These results are also in accordance with the study of Erdoğdu and Ekiz (2011) that demonstrated combining IR and UV resulted in the reduction of the bacterial load of total mesophilic aerobic bacteria inoculated on cumin. Similarly, a study conducted by Ha and Kang (2013) showed a synergistic bacteriocidal effect of combined IR and UV on red pepper inoculated with *E. coli*.

**Figure 7:**
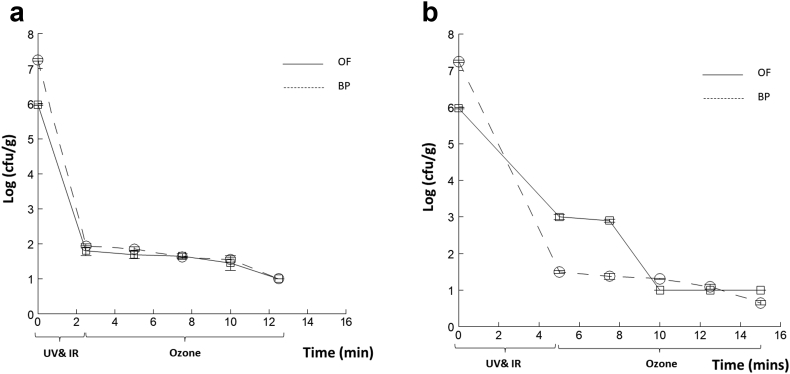
*E.coli* population kinetics of inoculated Onion Flakes (OF) and Black Pepper (BP), showing log (cfu/g), treated by combined treatment (UV & IR), for 2.5 min (a), and 5 min (b) followed by Ozone 2.5, 5, 7.5, 10 minutes. Error bars, SD, n =2.

To investigate the effect of the treatment order, a reduced exposure time of 2.5 and 5 min (UV and IR) was used, followed by 2.5, 5, 7.5 and 10 min for OF, with the results shown in Figure 7a and b, respectively.

With 2.5 min combined UV and IR treatment, the response for BP and OF were similar with 10 min of ozone treatment. This was the same with Figure 6a, with ozone first, for OF, but there was complete inactivation earlier with BP (after 7.5 min of UV and IR). With 5 min of UV and IR treatment, BP was more effectively decontaminated than OF, after the final treatment inactivation was complete for both samples. It is seen that the data points for OF in Figure 7 for UV and IR at 2.5 (a) and 5 min (b) show a slight difference; this cannot be explained directly but may be due to experimental error; what is clear, however, is that the sequential ozone treatment gave an improved performance when the UV and IR combined treatments were longer. This is evidenced by the log reduction. Interestingly, the treatment with the ozone for 2.5 and 5 min (Figure 6) are relatively close to each other, although the 5 min treatment is slightly lower. What is interesting however, is the impact of the subsequent treatments (UV and IR), which are much greater for the longer duration of ozone treatment. It is clear then that the longer treatment is having a beneficial effect.

**Figure 8:**
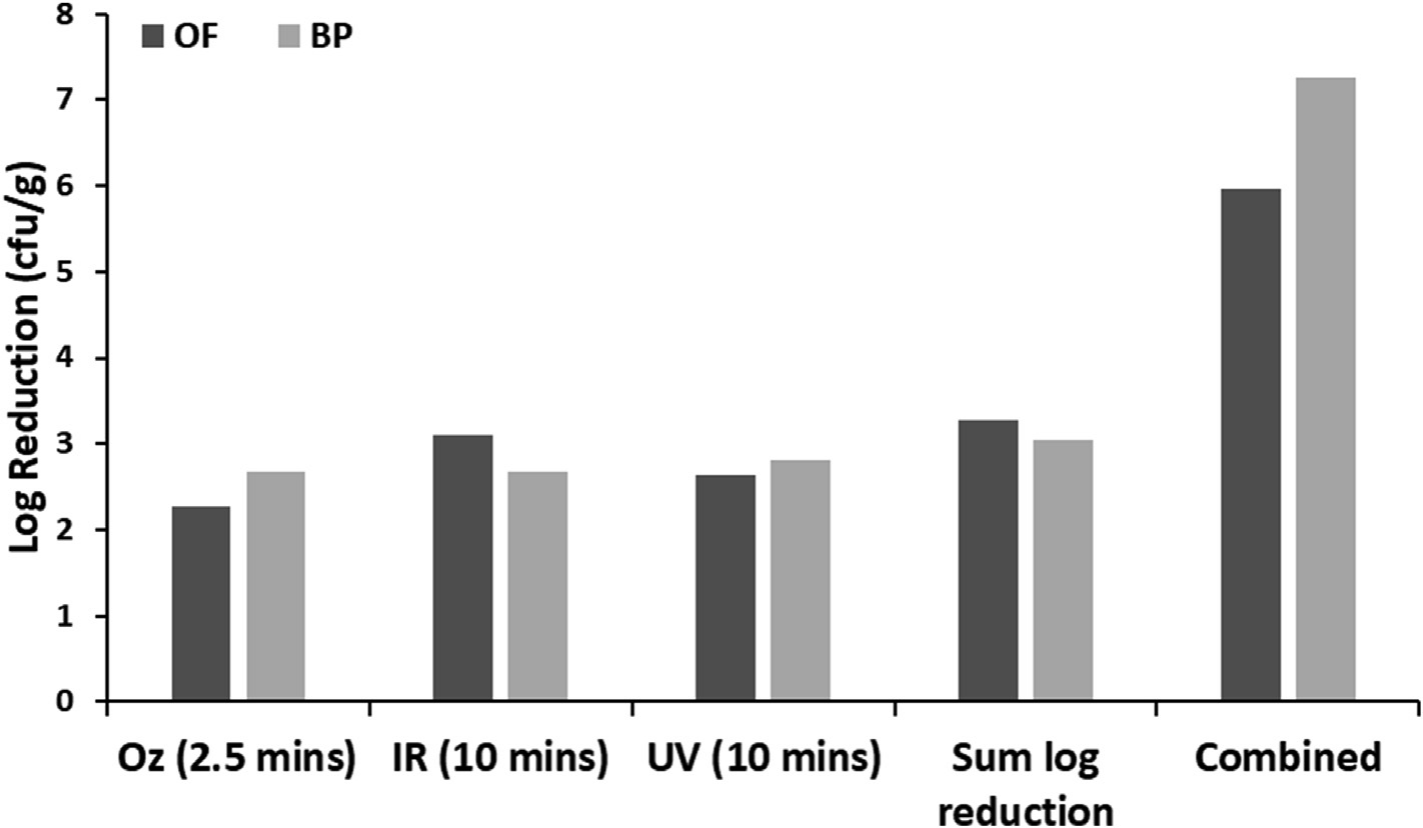
Comparison between additive (sum log reduction) and Combined effects of Ozone for 2.5 min, followed by simultaneous UV and IR treatment for 10 mins for OF (black, left columns) and BP (grey, right columns).

When comparing the data for additive or synergistic effects, the effects are dominated by the initial contamination levels for the combined treatments (i.e. with ozone, UV and IR in any order), and as high levels of inactivation were observed the differences are small for the treatment times chosen. As an example of the additive vs synergistic effects, Figure 8 shows the log reduction for OF and BP with Ozone treatment for 2.5 min, then IR and UV combined for 10 min. These single observed reductions were added (as shown in Sum log reduction) and compared to the combined effect of ozone (2.5 min), followed by simultaneously combination of UV and IR for 10 min. Clearly the combined system has enhanced the overall inactivation, with a difference in the log reduction of 2.69 for OF and 4.20 for BP. These results were similar for each of the combined treatments (data not shown).

### 3.5 Statistical analysis

Interestingly, there was no statistical difference (F-test, p < 0.05) between the datasets for the single treatments of Ozone and UV on OF and BP, however there was a statistical difference (p = 0.009) for IR. This may indicate that the treatment is independent of the surface colour and surface features for the treatment durations chosen for UV and Ozone, whereas there are differences for the IR, however, this will require deeper investigation. When comparing the single treatments against each other (t-test), it was found that there was no statistical difference for BP for any of the treatments, whereas for OF there was a significant difference (p < 0.05) between ozone and IR (0.0096), and UV and IR (0.0094).

For the analysis of the combination treatments there was no statistical differences found between any of the sets of data. This is likely more a reflection on the treatment values chosen and the narrow range of investigation but it does perhaps identify that the combined effect is limited in the current case, at least to p < 0.05 significance, even though some advantages seem evident.

## 4 Conclusion

This study has further investigated a novel method of combining Ozone, UV and IR by determining its efficacy on decontamination of Onion Flakes and Black Pepper. Response differences to the treatment were shown among the two types of spices, with better effectiveness for BP compared to OF. Ozone and UV treatment reduced completely the artificial bioburden (9.5 × 10^5^ cfu/mL) in <20 min, whereas for IR treatment this was achieved in around 30 min. Sequential ozone and combined UV and IR treatment gave improved results with 99.99% inactivation of *E. coli* over a reduced time. Starting with Ozone (2.5 and 5 min) or UV&IR (2.5 and 5 min) an improved performance was observed compared to the individual treatments alone. Greater analysis of the effect of these hurdle technologies and their combination on the organoleptic characteristics of the spices should be conducted in the future.

## Declarations

### Author contribution statement

Nada El Darra, Ian Watson: Conceived and designed the experiments; Performed the experiments; Analyzed and interpreted the data; Wrote the paper.

Fei Xie: Conceived and designed the experiments; Performed the experiments; Analyzed and interpreted the data.

Prashant Kamble, Zakir Khan: Conceived and designed the experiments.

### Funding statement

This work was supported by 10.13039/501100000266EPSRC (EP/M01343X/1), United Kingdom, and the Intramural grant, Beirut Arab University, Lebanon.

### Data availability statement

Data will be made available on request.

### Declaration of interests statement

The authors declare no conflict of interest.

### Additional information

No additional information is available for this paper.
